# Comparative Experimental Study of Wound Healing in Mice: Pelnac versus Integra

**DOI:** 10.1371/journal.pone.0120322

**Published:** 2015-03-23

**Authors:** Ana Carolina Câmara Wosgrau, Talita da Silva Jeremias, Dilmar Francisco Leonardi, Maurício José Pereima, Gabriella Di Giunta, Andrea Gonçalves Trentin

**Affiliations:** 1 Departamento de Biologia Celular, Embriologia e Genética, Centro de Ciências Biológicas, Universidade Federal de Santa Catarina Florianópolis, Santa Catarina, Brasil; 2 Serviço de Pediatria, Hospital Universitário, Universidade Federal de Santa Catarina, Florianópolis, Santa Catarina, Brasil; 3 Departamento de Pediatria, Centro de Ciências da Saúde, Universidade Federal de Santa Catarina, Florianópolis, Santa Catarina, Brasil; 4 Serviço de Anatomia Patológica, Hospital Universitário, Universidade Federal de Santa Catarina, Florianópolis, Santa Catarina, Brasil; 5 Departamento de Cirurgia, Universidade do Sul de Santa Catarina, Palhoça, Santa Catarina, Brasil; 6 Hospital Infantil Joana de Gusmão, Florianópolis, Santa Catarina, Brasil; UNIFESP Federal University of São Paulo, BRAZIL

## Abstract

Strategies for skin regeneration have been developed to provide effective treatment for cutaneous wounds and disease. Dermal substitutes have been used to cover the lesion to facilitate cell colonization, thereby promoting dermal regeneration. However, very little is known about Pelnac matrix especially at histological level. Therefore, the present work carried out an experimental *in vivo* comparative analysis between Pelnac and Integra, the most used dermal templates, in a mouse model of full-thickness skin wounds. Histological sections performed at the 3^rd^, 6^th^ and 9^th^ days after surgery were analyzed with regard to inflammatory response and vascularization. Both templates were completely incorporated in all animals at the end of the analyzed period. Pelnac-treated animals displayed reduced granulation tissue during the first 6 days of treatment compared to the animals treated with Integra at the same time period. The number of inflammatory cells (neutrophils) was similar in both groups during the period, significantly reducing at the end of inflammatory phase (9^th^ day of treatment) consistent with the progression of healing process. In addition, the density of blood vessels was also statistically similar in both matrices. Therefore, the two dermal templates displayed comparable biological behavior in tissue repair. It is noteworthy that this is the first experimental study comparing Pelnac and Integra dermal templates with focus on full-thickness skin wounds.

## Introduction

Skin, the largest organ of the human body, plays a crucial role in the protection against microorganisms, maintenance of body temperature and detection of sensory information about the external environment. Acute trauma, chronic wounds or surgical intervention can result in skin loss. Full-thickness injuries are characterized by the complete destruction of epithelial-regenerative elements. The healing occurs mainly by contraction from the edge of the wound, which may lead to cosmetic and functional defects (reviewed in [1]).

The healing process involves three overlapping phases (inflammation, tissue formation, and tissue remodeling) to achieve the tissue integrity and homeostasis [2]. In the inflammation phase, the granulation tissue is composed predominantly of inflammatory cells, mainly neutrophils that are recruited to the wound site and removed during the progression of the repair process. In the tissue formation phase, however, it consists of endothelial cells, macrophage and fibroblasts that begin to fill the wound area to restore tissue integrity [2, 3]. Moreover, full-thickness skin wounds of more than 1 cm in diameter require skin grafting as they cannot epithelialize on their own and may result in extensive scarring with limitations in mobility and severe deformities [4].

Great efforts have been made in order to create biomaterials for skin substitution. Hence, acellular dermal substitutes are very valuable for the wound bed preparation and the resultant recipient surface for effective graft integration. Dermal substitutes restore a full-thickness cutaneous wound and improve the quality of wound healing [1, 5]. They have proved to be suitable for many reconstructive surgical procedures [6, 7] as they serve as scaffolding for the ingrowth of fibroblasts and endothelial cells, which contribute towards neodermis formation [6].

Integra (Integra Life Sciences Corp., Plainsborg, NJ) is currently the most accepted dermal regeneration template to treat full-thickness skin wounds such as in burn patients [7–12]. Originally designed by Yannas and Burke (1980), it is a bilayer membrane system in which the deep layer (80 μm diameter mean pore size) is composed of bovine type I collagen fibers attached to shark chondroitin 6-sulfate glycosaminoglycan bounded to a temporary epidermal substitute layer of silicone [12]. Experimental studies in animal models of full-thickness skin wounds have histologically evaluated the host tissue integration, cellular infiltration and vascularization with the use of Integra [13–15]. When compared with another dermal templates, Integra provided results similar to Matriderm [13] and to Alloderm, Dermagraft and Dermalogen [14], but some differences were observed when compared with AlloDerm, DermACELL and DermaMatrix [15].

Similarly to Integra, Pelnac (Gunze Corp., Osaka, Japan) is a bilaminar membrane with a superficial silicone film layer and a porcine collagen sponge layer derived from pig tendon with pore diameter in the range 60–110 μm [16]. The use of this dermal template has been reported in skin reconstructive procedures [17–21]. Long-term followed-up studies of the use of Pelnac in clinical treatment of full-thickness skin defects revealed good results [18, 20]. The successful use of this dermal substitute in the reconstruction of cutaneous wounds secondary to cryoglobulinaemia was also described [17]. Though the clinical results of Pelnac have been reported, its use in the healing of full-thickness skin wounds has not been investigated at histological level.

In a recent article, we investigated the interaction of human skin-derived mesenchymal stroma cells with Integra and Pelnac dermal templates in a three-dimensional culture system. Both dermal substitutes equally supported the adhesion, spread and growth of theses cells, as well as their phenotypic profile [22]. However, despite the similarity in the structure and in the clinical use, the efficacy of these two dermal substitutes *in vivo*, in the regeneration of full-thickness skin wounds has not been investigated. Therefore, the aim of the present study was to assess histologically the host tissue integration of these two dermal templates and evaluate their effects in the vascularization and immune response of full-thickness skin wounds in mouse model. These data provide important information for the clinical use of these products and for further researches concerning the development of new therapeutic strategies for skin regeneration in order to provide effective treatment for cutaneous wounds and disease.

## Materials and Methods

### Animals

C57BL/6 mice (2-month-old, male and female, body weight 20–40 grams) were individually housed. All animals had free access to standard chow and drinking water and were maintained on a 12-h light/dark cycle. This study was carried out in strict accordance with the recommendations in the Guide for the Care and Use of Laboratory Animals of the National Institutes of Health. The protocol was approved by the Committee on the Ethics of Animal Experiments of the University of Santa Catarina (CEUA/UFSC Permit Number: 331-2009). All surgery was performed under Ketamine/Xylasine anesthesia, and all efforts were made to minimize suffering.

### Surgical procedure

Surgical procedures were aseptically performed in the animal facility operating rooms as previously described [23] with minor modifications. Briefly, after shaving, a full-thickness skin wound (10 mm x 10 mm) was excised from the dorsum of C57BL/6 mice. Segments of the dermal substitutes Integra (Integra Life Sciences Corporation, Plainsboro, NJ) or Pelnac (Pelnac Standart type, Gunze Limited, Kyoto, Japan) were sutured to the adjoining skin and underlying muscle with 6.0 nylon suture. No dressing was applied. Animals were randomly divided into two groups, with 15 mice per group. The first group received Integra and the second group was treated with Pelnac. Three, six and nine days after the surgery, five mice from each group were euthanized, the transplant area was excised, halved, fixed and embedded in paraffin for histological procedures as described below.

### Histological procedure

Excised tissues were fixed in 10% formalin solution for five days. All tissues were processed by using conventional histochemical techniques, embedded in paraffin wax and then sectioned at 3 μm thicknesses, mounted on glass slides, deparaffinized and stained with Haematoxylin–Eosin (HE). The density of inflammatory cells and blood vessels density in the dermis were stereological analyzed using the M-42 test system (Weibel No. 2, Tonbridge, England) (400×) that consists of 21 line segments (d) and 42 points in a testing area of 36.36 d^2^ [24]. The numerical density per area (NA) [25] of inflammatory cells or blood vessels was determined in a 45 μm^2^ frame. Random fields of the tissue were counted for each section. Images were taken with an optic microscope (BX41, Olympus) and digital sight camera (QC color, 3C Q-Imaging). The granulation tissue was assessed as the increase in skin thickness (μm) measured in the largest portion of each wound. The analyses were carried in a blinded manner.

### Statistical analysis

Values were expressed as the mean ± SEM with the indicated numbers (n) of experiments. Kolmogorov-Smironov test was used to assess the normality. Unpaired two-tailed t-test was used to determine significance. Statistical analyses were performed with GraphPad Prism 4 (Graph Pad Software, La Jolla, CA). Values of p < 0.05 were considered significant.

## Results

All animals survived and no complications, including infection, related to the procedure were observed. Nine days after transplantation, a complete integration of both templates, Integra and Pelnac, into the mouse tissue was observed in all animals.

### Inflammatory response

**Fig 1:**
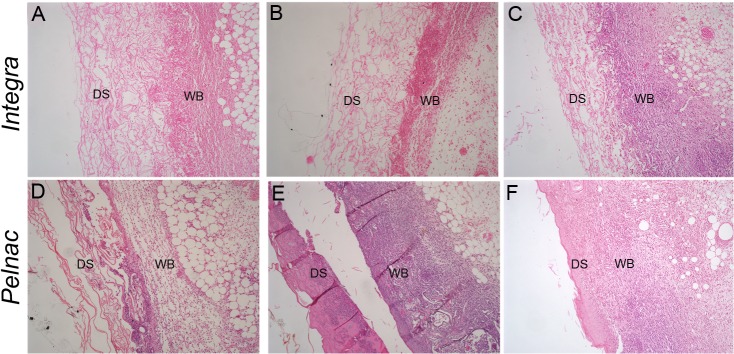
Histological aspects of wounds treated with dermal substitutes. HE-stained transversal sections of a full-thickness skin wound treated with (A-C) Integra or (D-F) Pelnac collected at (A, D) 3, (B, E) 6 and (C, F) 9 days after the procedure show a clear distinction between the dermal substitute (DM) and the wound bed (WB). Original magnification 100X.

**Fig 2:**
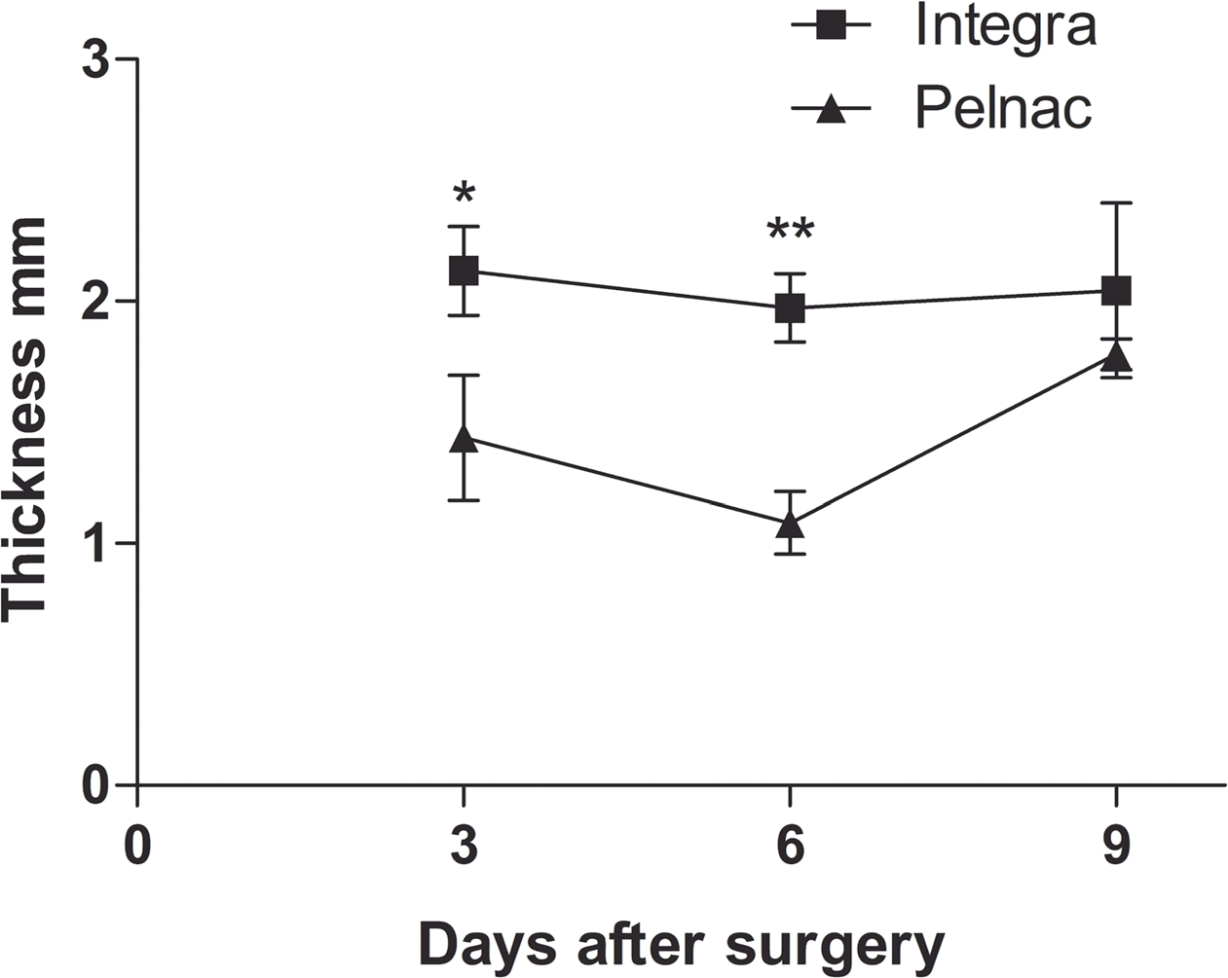
Analysis of the granulation tissue. The average thickness was measured in μm in the large portion of the granulation tissue of each field and results are expressed as the mean ± SEM of 5 animals. * p = 0.03 and ** p = 0.001 *vs*. Pelnac by two-tailed unpaired t-test.

Fig. 1 shows representative pictures of histological sections of wounds stained with HE during the nine days following treatment with dermal templates. Stereological analysis revealed that the thickness of granulation tissue in Integra-treated wounds remained constant during the period measuring 2.1, 1.9 and 2.0 mm at the 3^rd^, 6^th^ and 9^th^, respectively (Fig. 2 and S1 Table). In Pelnac-treated wounds, however, the granulation tissue was significantly reduced at the 3^rd^ (1.5-fold-reduction) and 6^th^ days (1.8-fold-reduction) after surgery, respectively. Similar values between the two animal groups were observed at the 9^th^ day after surgery.

**Fig 3:**
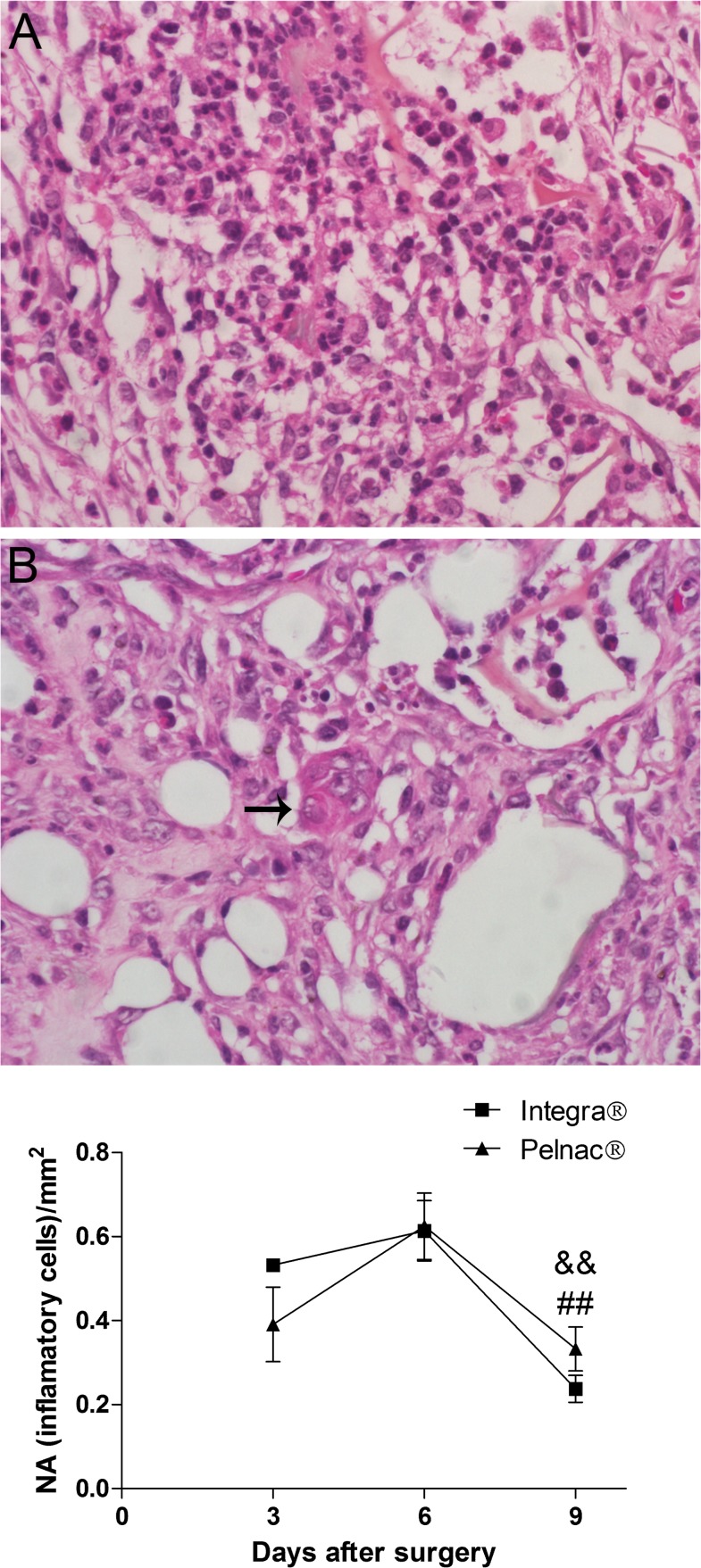
Histological aspects of wounds treated with dermal substitutes showing inflammatory cells. (A) Representative picture of HE-stained transversal section of a full-thickness skin wound. (B) Foreign-body giant cell (arrow). (C) Quantification of inflammatory cells during the 9 days after the surgical procedure. *NA* numerical density that represent the number of inflammatory cells per mm^2^. Data are expressed as the mean ± SEM of 5 animals. Not significant differences between the two templates were observed at any time point. && p = 0.001 Integra at day 6 *vs*. Integra at day 9 and ## p = 0.004 Pelnac at the day 6 *vs*. Pelnac at day 9 by two-tailed unpaired t-test. Original magnification 400X.

The density of inflammatory cells in the wound was investigated (Fig. 3). Neutrophils were predominant, corresponding to more than 95% of inflammatory cells in both dermal substitutes (Fig. 3A and S2 Table). In Integra-treated wounds, the number of neutrophils was similar between the 3^rd^ (0.5 neutrophils/mm^2^) and 6^th^ (0.6 neutrophils/mm^2^) days after surgery, significantly decreasing (2.6-fold-reduction) at the 9^th^ day (0.2 neutrophils/mm^2^) (Fig. 3C). Similar values of infiltrating neutrophils were observed in Pelnac-treated wounds during the period (0.4, 0.6 and 0.3 neutrophils/mm^2^ at days 3, 6 and 9, respectively). A significant reduction in this cell number was also found at the day 9 comparing to the day 6 (1.9-fold-decrease). In addition, very few foreign-body giant cells were observed at the day 9 of treatment (1–2 cells in the wound in each condition) in both dermal substitutes-treated animals (Fig. 3B).

### Vascular density

**Fig 4:**
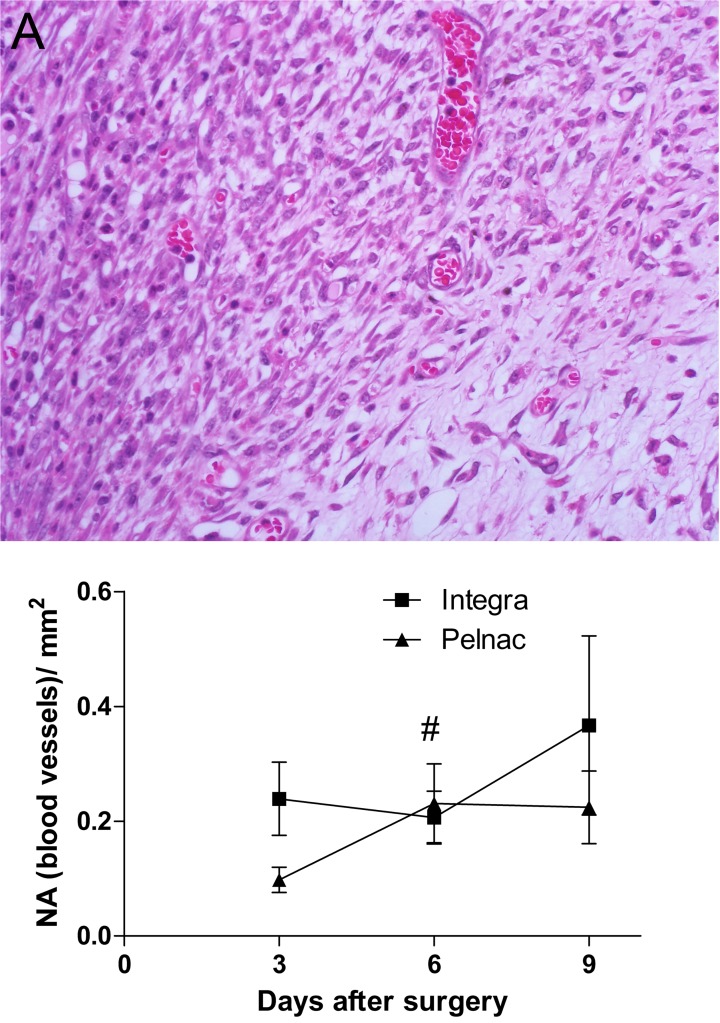
Histological aspects of wounds treated with dermal substitutes showing vascular density. (A) Representative picture of HE-stained transversal section of a full-thickness skin wound with emphasis in blood vessels. (B) Quantification of blood vessels during the 9 days after the surgical procedure. *NA* numerical density that represent the number of blood vessels per mm^2^. Data are expressed as the mean ± SEM of 5 animals. Not significant differences between the two templates were observed at any time point. # p = 0.05 Pelnac at the day 3 *vs*. Pelnac at day 6 by two-tailed unpaired t-test. Original magnification 400X.

The hematoxylin and eosin staining also showed the capillary network during the experimental analysis (Fig. 4A). Results revealed constant density of blood vessels in Integra-treated animals through the analyzed period (0.24, 0.20 and 0.36 blood vessels/mm^2^, at days 3, 6 and 9, respectively) (Fig. 4B and S3 Table). On the other hand, a very reduced number of blood vessels (0.09 blood vessels/mm^2^) was observed in Pelnac-treated animals at the 3^rd^ day after surgery. This value significantly increased at the 6^th^ day (0.23 blood vessels/mm^2^) and remained constant at the 9^th^ day (0.22 blood vessels/mm^2^) after surgery (Fig. 4B). Although at the day 3 the value was 2.6-fold less than in the Integra-treated wounds in the same time, it was not statistically significant. The number of blood vessels was also similar between the two matrices at the 6^th^ and 9^th^ days.

## Discussion

Though the clinical use of Pelnac in reconstructive procedures have been reported, its has not been investigated at histological level [1]. This is the first experimental study concerning the histological evaluation of skin lesions treated with Pelnac. In the present work, we have comparatively analyzed the Integra and Pelnac dermal substitutes in a murine model full-thickness skin wounds. Despite some differences, both matrices gave similar results at the end of the analyzed period concerning the integration in animal tissues, inflammatory response and vascularization. Further studies of the long-term evaluation of these animals and the activation/rest infiltrating cells state would be important to confirm these findings.

Regarding the healing process, in our experiments, Integra-treated animals displayed higher values of granulation tissue than Pelnac-treated animals during the first 6 days of treatment. At the 9^th^ day after surgery, however, the thickness of granulation tissue was similar between the two matrices, when the inflammation decreases and the second phase of healing process (tissue formation) take place. Therefore, Integra seems to favor the formation of granulation tissue in the initial phase of the wound healing. However, at the end of the period it is similar in both matrices.

Consistent with the evolution of the healing process, neutrophils were the predominant inflammatory cell observed in our experiments with increased values in the first analyzed time points (3^rd^ and 6^th^ days after surgery), significantly decreasing thereafter (9^th^ day after surgery). This profile was similar in animals treated with both matrices. For a successful repair, the resolution of the inflammatory response is essential and excessive or prolonged inflammatory response results in increased tissue injury and poor healing [2]. Our results, therefore showed that, despite the enhanced granulation tissue observed in Integra-treated animals in the initial period of treatment, both matrices contributed similarly to a proper inflammatory response during the healing process.

Foreign-body giant cells are also related to the inflammatory response and are most commonly observed at the interface of tissue and implanted medical devises, prostheses and biomaterials [26, 27]. A previous study reported numerous foreign-body giant cells in mice treated with Integra that was associated with the cross-linking of the collagen bovine fibers of the scaffold [14, 26, 28]. Despite that, another histological study reported the perfect integration of Integra after 5 years of implantation [29]. In our experiments, however, a very small number of them (1–2 in the whole lesion) were observed at the 9^th^ day after surgery in both Integra- and Pelnac-treated animals consistent with the remodeling and deposition of the extracellular components further suggesting that the tissue formation phase take place this time [2, 3]. The minor quantity of these foreign-body giant cells is also consistent with the complete integration of both matrices in the mouse tissue observed in our study and corroborates the contribution of both matrices to the resolution of inflammatory response.

Neovascularization is another important event that takes place in the second stage of the wound repair [3]. In our results, similar density of blood vessels was observed in Integra- and Pelnac-treated animals, indicating that both matrices allow neovascularization. Although not significant, the 2.5-fold and 1.6-fold increase in blood vessel numbers of Integra-treated animals at the 3^rd^ and 9^th^ days after surgery, respectively, compared to Pelnac-treated animals at the same time points, might indicate a possible improved efficiency of this dermal template in neovascularization. Moreover, Spazzapan and colleagues (2014) demonstrated improved peripheral tissue oxygenation of diabetic feet with the use of Integra associated with split thickness skin graft compared to the reconstruction with the graft only [30]. Further studies, especially those associating other elements of tissue engineering, such as cells and/or growth factors, would be useful to corroborate this finding.

Taken together, our results revealed that, despite some differences, both Integra and Pelnac dermal templates display similar biological behavior in wound healing process in a mouse model of full-thickness injury. This finding corroborate our previous results in which Integra and Pelnac were equally efficient in support the growth of skin-derived mesenchymal stromal cells in a three-dimensional culture system [22]. Common features between Integra and Pelnac could explain these results. Both have a bilayer composition with an external temporary epidermal silicone sheet, and a dermal component made of collagen [12, 16]. Nevertheless, Pelnac collagen dermal component is derived from pig tendon [16], while the porous dermal component of Integra is made of cross-linked bovine type I collagen fibers and chondroitin-6-sulfate [12]. Moreover, bovine and porcine collagens were demonstrated to display similar characteristics for the production of scaffolding materials [31].

## Conclusions

This is the first experimental study to compare Integra and Pelnac dermal templates with focus on full-thickness skin wounds. This is also the first report concerning the histological evaluation of skin lesions treated with Pelnac. Our data showed that, despite some differences in the granulation tissue thickness, inflammatory response and the blood vessel density are similar in wounds treated with Pelnac and Integra, suggesting that both dermal substitutes have a similarly favorable biological behavior and could be successfully implanted.

## Supporting Information

S1 Table Thickness of the granulation tissue. Data of Fig. 2. Values represent the individual measures and means expressed as mm, standard errors (SEM), the number of animal (n) and the P value between the animal groups.
(XLSX)

S2 Table Quantification of inflammatory cells. Data of Fig. 3C. Values represent the individual measures and means expressed as inflammatory cells/mm^2^ standard errors (SEM), the number of animal (n) and the P value between the animal groups.
(XLSX)

S3 Table Quantification of blood vessels. Data of Fig. 4B. represent the individual measures and means expressed as blood vessels/mm^2^ standard errors (SEM), the number of animal (n) and the P value between the animal groups.
(XLSX)
